# Platinum-based chemotherapy in metastatic prostate cancer: what possibilities?

**DOI:** 10.1007/s00280-023-04604-w

**Published:** 2023-11-07

**Authors:** Martina Catalano, Andrea Lapucci, Stefania Nobili, Irene De Gennaro Aquino, Ismaela Anna Vascotto, Lorenzo Antonuzzo, Donata Villari, Gabriella Nesi, Enrico Mini, Giandomenico Roviello

**Affiliations:** 1 Department of Health Sciences, Section of Clinical Pharmacology and Oncology, University of Florence, 50139 Florence, Italy; 2 Department of Neuroscience, Psychology, Drug Research and Child Health, University of Florence, 50139 Florence, Italy; 3 School of Human Health Sciences, University of Florence, 50134 Florence, Italy; 4 Department of Experimental and Clinical Medicine, University of Florence, 50134 Florence, Italy; 5 Department of Health Sciences, Section of Pathological Anatomy, University of Florence, 50139 Florence, Italy; 6 University of Florence, Viale Pieraccini 6, 50134 Florence, FI Italy

**Keywords:** Metastatic prostate cancer, Platinum-based chemotherapy, DNA damage repair, PARP inhibitors, Combination therapy

## Abstract

Metastatic prostate cancer is a major health burden worldwide, necessitating the continuous development of effective treatment strategies. Androgen deprivation therapy remains the cornerstone of prostate cancer treatment, but novel approaches are needed for metastatic castration-resistant prostate cancer (mCRPC). Recent studies have highlighted the prevalence of mutations in DNA repair genes, including *BRCA1* and *BRCA2*, in mCRPC patients, rendering them more susceptible to platinum-based chemotherapy and Poly (ADP-ribose) polymerase (PARP) inhibitors. Platinum-based chemotherapy, particularly in combination with taxanes, has demonstrated encouraging activity in mCRPC, as well as homologous recombination gene alterations have shown increased sensitivity to platinum compounds in these patients. The combination of platinum-based chemotherapy with PARP inhibitors represents a novel and potentially effective therapeutic strategy for this subgroup of patients. However, the optimal sequence of administering these agents and the potential for cross-resistance and cross-toxicities remain areas requiring further investigation. Prospective randomized studies are essential to elucidate the most effective treatment approach for this challenging patient population. This review aims to explore the potential of platinum-based chemotherapy in the context of prostate cancer, and more in detail in homologous recombination repair (HRR) mutated patients. We discuss the synergistic effects of combining platinum compounds with PARP inhibitors and the potential benefits of adopting specific therapeutic sequences.

## Introduction

Despite notable progress in the development of treatment strategies during the past decade, prostate cancer (PC) remains the most prevalent malignancy and the second leading cause of cancer-related mortality in men worldwide [1]. PC exhibits a spectrum of clinical behaviors, spanning from indolent, slowly developing tumors to aggressive, rapidly advancing forms. Approximately 5–10% of PC patients receive a diagnosis of metastatic disease, and their prognosis is unfavorable, with a 5-year survival rate hovering around 30% [2]. The biology of PC encompasses a range of intricate processes, including hormonal regulation, genetic and molecular alterations, and interaction with various components of tumor microenvironment. A comprehensive understanding of the biology of PC is essential to untangle the complexities involved in its onset, progression, and response to treatment. This dynamic field continues to evolve, offering hope for more effective and tailored therapies for PC patients.

However, androgen-deprivation therapy (ADT) continues to represent the cornerstone of PC treatment. Docetaxel and anti-androgens such as enzalutamide, apalutamide, darolutamide, and abiraterone, as monotherapy or in combination, are approved for the treatment of metastatic hormone sensitive PC; while, the therapeutic scenario of metastatic castration-resistant PC (mCRPC) has been considerably enriched by the introduction of abiraterone and enzalutamide, cabazitaxel, immuno-modulatory agent sipuleucel-T, radiopharmaceutical agents such as radium-223 (only in case of bone metastasis), and ^177^Lutetium-prostate-specific membrane antigen (PSMA)-617 [3]. Recent investigations have revealed that approximately 25% of patients with mCRPC harbor tumor somatic or germline mutations in DNA damage repair (DDR) genes including breast cancer susceptibility genes *BRCA1* and *BRCA2*, as well as other genes implicated in homologous recombination repair (HRR) [4]. These genetic alterations have been associated with an unfavorable prognosis in terms of both survival and disease progression [5]. Genomic aberrations affecting these genes, which lead to deficiencies in DNA damage sensing or repair, may increase the sensitivity of tumors to platinum-based chemotherapy as well as to Poly (ADP-ribose) polymerase (PARP) inhibitors (PARPis) [5–7]. Currently, the Food and Drug Administration (FDA) and the European Medical Agency (EMA) have approved two PARPis, olaparib and rucaparib, for the treatment of mCRPC patients [8–11]. Although platinum-based chemotherapy has demonstrated advantages in terms of palliative benefits, objective response, and progression-free survival (PFS) in phase II studies involving mCRPC, these improvements did not translate improved overall survival (OS) [12–15].

With emerging data indicating a high prevalence of somatic and germline alterations in DDR genes among patients with advanced PC, coupled with the efficacy of PARPis in this patient population, interest in platinum-based drug treatments has also been renewed. The hypothesis is that platinum-based therapy may exhibit higher efficacy in this specific subgroup of PC patients, as observed in individuals with other types of cancer such as triple-negative breast cancer (TNBC) [16]. In this comprehensive review, our objective is to summarize the current findings and explore potential future directions of platinum-based chemotherapy in the metastatic setting of PC.

## Platinum-based chemotherapy in prostate cancer

Platinum compounds exert their antitumor effects by forming covalent adducts with cellular DNA, inducing DNA damage during the G2 phase, and cell death [17]. Nonetheless, the literature suggests that only a fraction, possibly ranging from 1 to 10%, of intracellular cisplatin can ultimately penetrate the nucleus and initiate a reaction with DNA, leading to cell cycle arrest and apoptosis in rapidly proliferating tumor cells [18]. Cisplatin, the pioneer of platinum-based anti-cancer drugs, was initially discovered in the late 1960s and received approval for cancer treatment in 1978 [19]. Its therapeutic efficacy has been demonstrated in various malignancies, including ovarian, breast, and gastrointestinal cancers. However, despite its anti-tumor properties, the prolonged use of cisplatin is associated with non-specific therapeutic effects and systemic toxicity mainly represented by myelosuppression, neurotoxicity, nephrotoxicity, and ototoxicity, leading to significant damage to normal tissues [17, 20]. The other two clinically approved platinum drugs, carboplatin and oxaliplatin, show different activity and toxicity profiles compared with cisplatin.

Platinum compounds are administered intravenously. However, the efficacy of these compounds as single agents in unselected patients has generally been moderate, and some combination therapies have led to significant toxicity. In the context of PC, platinum compounds have been extensively studied both as monotherapy and in combination therapy [21]. However, most of these studies have involved small case series and have recruited patients without considering tumor molecular characteristics.

In a pooled analysis conducted by Leal et al., various studies investigating platinum-containing chemotherapy regimens for patients with CRPC were collected [22]. Overall, the data indicated a statistically significant benefit of platinum-based chemotherapy in terms of clinical overall response rate, but there was insufficient evidence to demonstrate or exclude improvements in PFS or OS. Notably, response rates were higher when platinum compounds combined with other cytotoxic agents were compared with platinums alone. It is worth mentioning that some of these studies were conducted prior to the approval of taxanes for the treatment of CRPC [22]. Also, data from a limited number of randomized trials included in this meta-analyses confirmed increased response rates for chemotherapy regimens containing platinum compounds compared to other regimens. However, specific patient populations, such as those with aggressive variants of PC or genomic defects in DDR pathways, seem to derive more substantial benefits from platinum-based treatments [22].

In the past, a phase III clinical trial (SPARC study) investigated the potential efficacy of satraplatin, an oral platinum-based compound in patients with mCRPC experiencing progression after one prior chemotherapy regimen [12]. Satraplatin combined with prednisone demonstrated improvements in PFS (*p* < 0.001) and pain control compared with prednisone plus placebo, but there was no OS benefit observed between the two arms (*p* = 0.80). Satraplatin was well tolerated, although myelosuppression and gastrointestinal disorders occurred more frequently compared to placebo [12]. To date, research on satraplatin in Western countries has been substantially stopped.

Taxane–platinum combinations have shown promising activity in mCRPC in single-group clinical studies, but not in randomized trials. For instance, the RECARDO trial, a randomized phase II trial comparing docetaxel alone versus docetaxel plus carboplatin in patients with CRPC who progressed after responding to prior docetaxel chemotherapy, did not reveal any differences in PFS or OS [23]. This inconsistency may be attributed to the previous docetaxel treatment as well as the lower dose of docetaxel used in combination with carboplatin and the use of docetaxel instead of cabazitaxel, the agent of choice in CRPC patients previously treated with a docetaxel-containing treatment regimen [24].

A more recent phase I/II randomized study evaluated the efficacy of cabazitaxel plus carboplatin in men with progressive mCRPC [15]. The addition of carboplatin to cabazitaxel showed improved clinical efficacy compared to cabazitaxel alone. At a median follow-up of 31.0 months, the combination therapy resulted in a median PFS of 7.3 months, compared to 4.5 months with cabazitaxel alone. Although adverse events were more common with combination therapy, it was generally safe and well-tolerated. The most common grades 3–5 adverse events were fatigue, anemia, neutropenia, and thrombocytopenia and no treatment-related deaths were reported [15]. These findings suggest that taxane-platinum combinations have a clinically beneficial role in advanced PC, although randomized phase III study should be planned to further investigate their efficacy.

## Platinum-based chemotherapy in HHR mutated prostate cancer

**Fig. 1:**
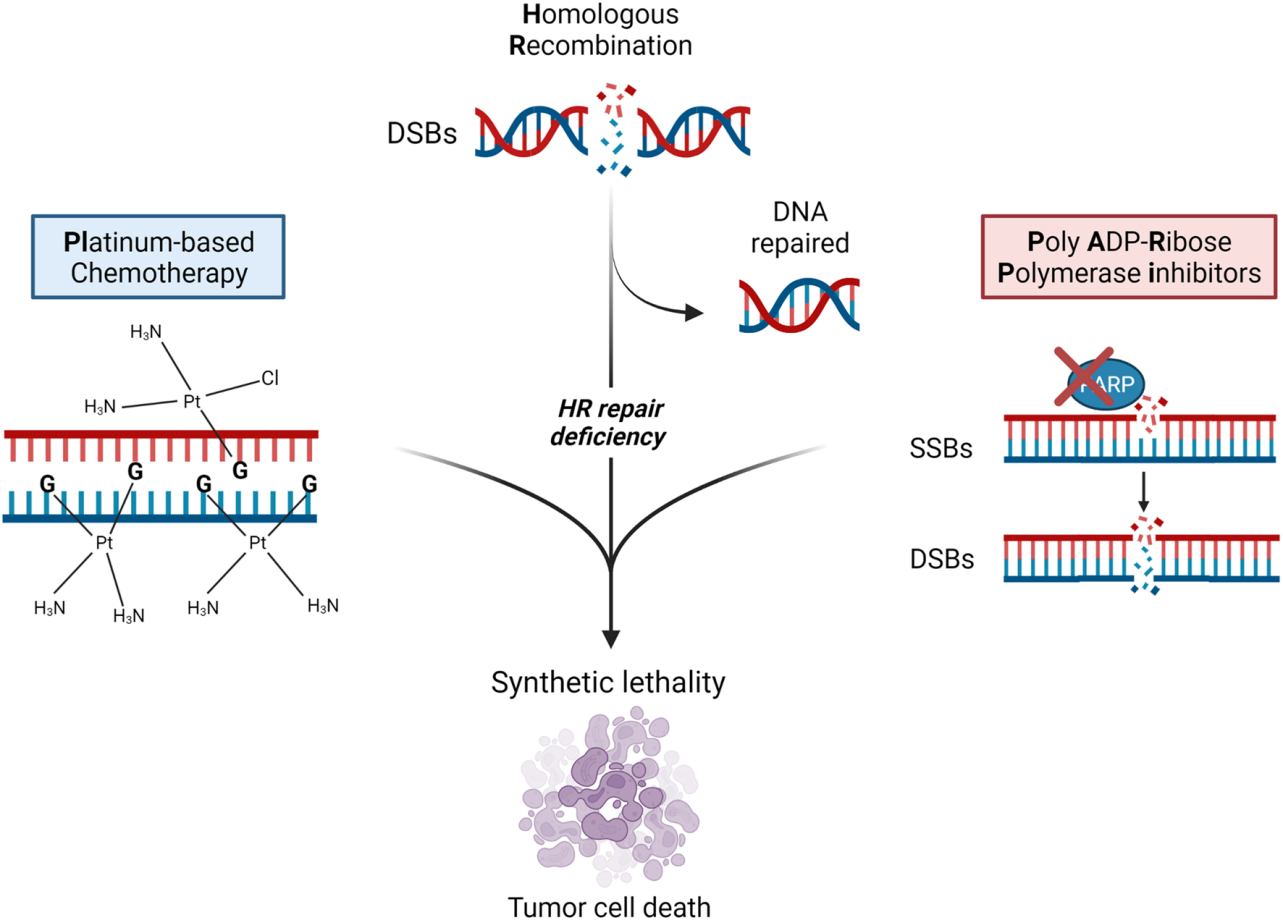
Synthetic lethality of platinum-based chemotherapy and PARP inhibitors in HR deficiency tumors**.** HR, homologous recombination; DSBs, double strands breaks; SSBs, single strands breaks. Created with BioRender.com

Genomic instability is a commonly observed characteristic of tumorigenesis, and impaired DNA repair is recognized as a fundamental feature of cancer development [25]. HRR is a DNA repair mechanism that specifically acts on DNA double-strand breaks (DSBs) and interstrand cross-links (ICL) [26]. Deficiencies in the HRR pathway have been linked to various tumor types, such as breast, ovarian, prostate, and pancreatic cancers. This deficiency is referred to as homologous recombination deficiency (HRD), while tumors exhibiting intact HRR are described as homologous recombination proficient (HRP) [27]. The presence of HRD in tumors can render them more susceptible to combined treatments with platinum drugs, that induce ICLs, and PARPis, resulting in synthetic lethality [25] (Fig. 1).

Genomic abnormalities impairing DNA repair genes are present in 20%–30% of advanced PC [6, 28, 29]. These actionable molecular alterations and aberrations in HR occur in a considerable fraction of localized PCs and, even more frequently in metastatic disease [30]. Some of these alterations, which can be germline or somatic, have been associated with sensitivity to platinum compounds and/or PARPis in both preclinical studies and clinical trials [31].

In TNBC, carboplatin has demonstrated high efficacy in patients with known tumors carrying variations in *BRCA1* and *BRCA2* [32]. Moreover, there is evidence that patients with variations in other HR genes, aside from *BRCA1* and *BRCA2*, can benefit from platinum-based treatment. Conversely, alterations in other non-homologous recombination DNA damage response genes, such as *PTEN*, do not result in a similar level of response [32]. Accumulated data in ovarian cancer have shown that the approach of assessing HR deficiency is a positive predictor of response to platinum-based drugs [33].

Encouraging anti-tumor activity of platinum-based chemotherapy in a patient with mCRPC and DNA repair gene defects (i.e., *BRCA1/2* mutations) has been found [34]. In a study by Mota et al., response to platinum-based chemotherapy was retrospectively assessed in patients with mCRPC who underwent somatic and germline genomic sequencing [35]. They found that prostate-specific antigen (PSA) responses occurred more frequently in patients with genomic alterations in DDR genes. Although, there was a trend toward longer time on treatment in the DDR-mutant group, no difference in OS was observed. Importantly, the analysis was limited to patients who received platinum-based chemotherapy after progressing on taxane therapy, where the response was more likely attributed to the platinum agent compared to platinum and taxane combination therapy [35]. These findings are not only consistent with previous reports of improved response of *BRCA*-altered tumors to platinum-based chemotherapy but also highlight responses in tumors with non-*BRCA* DDR gene alterations, including *PALB2, FANCA*, and *CDK12* [36–38]. This suggests that a broader DDR gene panel, including non- *BRCA* mutations, could be used to identify a higher number of mCRPC patients more likely to benefit from platinum chemotherapy, whether administered alone or combined with a taxane. Moreover, other clinical subsets of PC, described as aggressive variants, including those with low PSA expression, visceral metastasis, or histologic neuroendocrine differentiation, may also derive particular benefit from platinum chemotherapy, and the presence of a genomic alteration in a DDR gene is just one variable that could aid in patient selection for this therapy [39]. Like small cell lung cancer, neuroendocrine prostate cancer (NEPC) tends to exhibit an initial responsiveness to platinum-based chemotherapy, demonstrating objective response rates ranging from 50 to 60% [40, 41]. The mechanisms underlying the response of NEPC and aggressive variant prostate cancer (AVPC) to this treatment regimen may also be influenced by inherent tumor suppressor gene losses and/or aberrations in DNA repair pathways. The rational use of a combination chemotherapy involving cabazitaxel and carboplatin is particularly noteworthy in the context of NEPC, given the efficacy of cabazitaxel in CRPC and the frequent observation of mixed tumor histologies (comprising both adenocarcinoma and NEPC elements) are within the spectrum of NEPC [42]. The utilization of carboplatin in combination with cabazitaxel has garnered support from the National Comprehensive Cancer Network guidelines, as a viable option for patients exhibiting aggressive variant clinical characteristics or unfavorable genomic profiles, which may involve loss-of-function alterations in at least two of *PTEN*, *TP53*, and *RB1* [43].

In a multicenter retrospective analysis by Schmidt et al. involving 508 patients with mCRPC, encouraging antitumor efficacy was observed with platinum-based therapies in patients with tumors harboring DNA repair gene abnormalities [21]. Although, numerically higher rates of PSA level decreases and soft tissue responses were observed in patients with DNA repair gene abnormalities compared to those without, there was no statistically significant difference and no OS benefit. In the subgroup of 44 patients with *BRCA2* gene mutations, a PSA level decrease of at least 50% was documented in 23 patients (63.9%), and soft tissue responses were observed in 17 patients (38.6%) with evaluable disease [21]. In this study, the response to platinum-based monotherapy was comparable to the recently reported trials of PARP monotherapy in patients with DNA repair gene abnormalities [44–46]. However, the response to platinum-based combination therapy was more favorable than monotherapy, and in most cases, a taxane was chosen as the combination partner.

Fan et al. reported distinct responses to platinum-based chemotherapy in patients with and without DDR gene alterations and among mCRPC patients harboring alterations in different HR genes [47]. Of the 55 evaluated patients, 23 had genomic defects in HR pathway genes. The median PSA–PFS for the 23 patients with HR defects was 6.7 months, compared to 2.6 months for the 22 patients without HR defects (*p* = 0.001). Patients with somatic HR defects displayed a shorter PSA–PFS compared to those with germline HR defects (4.5 months *vs*. not reached). The PSA50 response rate (patients who survived for 12 weeks and had a PSA decline of over 50% from baseline) was higher in patients with *BRCA2* or *ATM* defects (75.0%) compared to those with *CDK12* defects (22.2%; *p* = 0.06). Overall, patients with *BRCA2* or *ATM* defects exhibited prolonged PSA–PFS compared to those with *CDK12* defects or other HR defects (*p* = 0.038) [47].

Recently, a systematic review and meta-analysis were conducted by Fazekas et al. to evaluate the effectiveness of various treatment modalities in patients with *BRCA*-positive mCRPC. Their findings indicated that both PARPis and platinum-based therapies exhibited similar rates of PSA50 response and OS outcomes. This underscores the suitability of platinum-based therapies as a viable treatment option for individuals with *BRCA*-positive mCRPC. Nonetheless, the need for prospective interventional studies comparing these therapeutic agents remains imperative to establish a more robust level of evidence [48].

## Platinum-based chemotherapy and PARP inhibitors

The efficacy of PARP inhibition relies on the presence of mutations or alterations in DNA damaged genes, particularly those involved in HR. The presence of HR gene mutations can enhance the amplification of DNA damage effects caused by platinum drugs, suggesting that PARPis could be effective like adjunctive therapy with cisplatin or carboplatin.

Since 2020, PARPis have emerged as a therapeutic option in metastatic PC. First, olaparib was approved for adult patients with suspected or confirmed germline or somatic HRR gene-mutated mCRPC, who had progressed following prior treatment with enzalutamide or abiraterone [49]. Then, rucaparib was approved for the treatment of adults, with mCRPC harboring deleterious *BRCA* germline and/or somatic mutations, who had received androgen receptor-directed therapy and one taxane [50].

Previous studies have demonstrated that combining PARP inhibition with cisplatin significantly increased lifespan and restored nerve conduction velocity in animal models [35]. PARPis can also provide protection against dose-limiting toxicity associated with certain anticancer therapies [37].

In ovarian cancer patients, the combination of PARP inhibition with carboplatin and paclitaxel has significantly improved PFS in a phase II trial [51]. Olaparib and paclitaxel combined with carboplatin can improve the serological indicators of patients with ovarian cancer, enhance disease control, and reduce the recurrence rate, with no extra toxic side effects [52]. Although the synergistic potential of administering PARPi and chemotherapy concurrently is appealing, its implementation in clinical practice has encountered significant challenges due to overlapping toxicities, particularly myelosuppression. Consequently, the combined approach of PARPi and chemotherapy in ovarian cancer has been discontinued.

In a phase II trial in advanced breast cancer, veliparib–carboplatin added to standard therapy resulted in higher rates of pathological complete response (51%) than standard therapy alone (26%) specifically in TNBC, with a greater toxicity than that of the control [53]. Likely, the addition of veliparib to cisplatin significantly improved PFS in patients with *BRCA*-like metastatic TNBC (5.9 *vs*. 4.2 months, *p* = 0.01), but not in patients with non-*BRCA*-like metastatic breast cancer (4.0 *vs*. 3.0 months, *p* = 0.57) compared to cisplatin plus placebo [54]. The addition of veliparib to a highly active platinum combination (carboplatin–paclitaxel) resulted in significant and durable improvement in PFS compared to chemotherapy alone (14.5 *vs.* 12.6 months, p < 0.001) in patients with germline *BRCA* mutation-associated advanced breast cancer [55].

A phase I study was conducted to evaluate the combination of veliparib with cisplatin and gemcitabine in patients diagnosed with advanced pancreatic cancer harboring germline *BRCA* mutations or with a family history of *BRCA*-related cancers [56]. The study enrolled nine and seven patients with or without *BRCA* mutations, respectively. Notably, seven patients with *BRCA* mutations displayed positive responses, with six achieving partial responses and one experiencing a complete response. However, it is essential to mention that the patient who achieved a complete response later developed acute myeloid leukemia (i.e., approximately 2.5 years into the treatment), likely associated with the therapy. No responses were observed in patients without *BRCA* mutations. Several phase II trials have been conducted to assess the effectiveness of PARPi in treating pancreatic cancer. Among these trials, a multicenter phase II study enrolled patients with pathogenic germline *BRCA1* or *BRCA2* mutations and recurrent solid tumors to evaluate olaparib monotherapy after first-line chemotherapy [55]. The encouraging results reported in pancreatic cancer patients offered a solid rationale to continue the development of PARPis for *BRCA*-related pancreatic cancer [57]. The subsequent phase III POLO trial demonstrated a longer median PFS in patients treated with olaparib compared to those who received placebo (7.4 months *vs.* 3.8 months), while maintaining quality of life. It is important to note that no significant difference in median OS was observed between the two groups (19.0 months *vs.* 19.2 months, respectively) [58].

To date, no data on the combination use of platinum-based chemotherapy and PARPi are available in mCRPC, and few sequence data are reported in the literature. Mota et al*.* in their translational study examined responses to platinum chemotherapy after progression on a PARPi in patients with *BRCA* and *ATM* mutations. It is unclear whether tumors that acquire resistance to PARPi can still respond to other DNA damage-targeting agents, including platinum chemotherapy. These authors found that three out of eight patients with DDR mutations (37%) obtained some clinical benefit from platinum-based chemotherapy after progression on a PARPi, with a patient achieving a radiographic partial response. However, outcomes in this advanced patient population were generally poor. Notably, their study included four patients with deleterious alterations in *ATM* who received platinum-based chemotherapy either before or after receiving a PARPi. None of these patients achieved a PSA50 response and all experienced rapid disease progression. Although this finding is based on a limited sample size and needs confirmation in larger studies, it highlights the need for novel therapeutic approaches for approximately 4% of mCRPC patients who have deleterious alterations in *ATM* [28].

Some authors have reported the incorporation of PARPi and platinum-based chemotherapy in the treatment history of mCRPC, showing encouraging efficacy results [34].

Recently, Slootbeek et al. investigated the cross-resistance between platinum-based chemotherapy and PARPi in mCRPC patients with HRR mutations [59]. The analysis unveiled that the sequence in which these therapeutic agents were administered mainly impacted on the median PFS associated with platinum-based chemotherapy. Specifically, when PARPi was administered as the initial treatment (i.e., prior platinum-based chemotherapy), a reduction by 3.6 months was observed in the PFS of platinum-based chemotherapy… Conversely, the median PFS of PARPi administered after platinum-based chemotherapy was only 0.9 months shorter than the median PFS when PARPi was administered as initial treatment. Regarding response rates among patients who received PARPi as the initial treatment, 37.5% exhibited a > 50% decline in PSA levels in response to subsequent platinum-based chemotherapy, and 25.0% showed a radiographic response. In contrast, for those who received platinum-based chemotherapy initially, 60.0% experienced a > 50% decline in PSA levels, and 55.6% showed a radiographic response to subsequent PARPi therapy [60]. These observations imply that starting treatment with platinum-based chemotherapy might result in a lower development of cross-resistance to PARPi when compared to the opposite treatment sequence (PARPi followed by platinum-based chemotherapy). Nonetheless, the precise mechanisms of resistance underlying these findings remain to be fully elucidated. Consequently, the acquisition of additional data on resistance mechanisms will be of utmost importance in defining the most optimal treatment sequence for mCRPC patients with HRR mutations in the future. More information on cross-resistance will be derived from the ongoing phase II COBRA trial, which compares carboplatin and olaparib head-to-head with a cross-over design in mCRPC patients (NCT04038502), although in women’ cancers, a phase I/Ib trial by Lee et al. examined the impact of different drug administration sequences (i.e., olaparib followed by carboplatin and carboplatin followed by olaparib) on olaparib pharmacokinetics and platinum–DNA adducts in peripheral blood mononuclear cells as pharmacodynamic measures [61]. Their findings revealed that administering olaparib before carboplatin led to a reduction in carboplatin cytotoxicity. Conversely, when carboplatin was given first, it caused an accumulation of intracellular olaparib, thereby reducing the availability of bioactive olaparib. These results suggest that administering carboplatin prior to olaparib may be more beneficial, indicating that the order of drug administration could potentially optimize the clinical benefits.

Some clinical trials are currently investigating the effectiveness of platinum-based chemotherapy alone or in combination with other agents in mCRPC patients (Table 1).

**Table 1:** Ongoing clinical trials with platinum-based chemotherapy in mCRPC

Study	Setting	Treatment	Status	Estimated enrollment	Phase	Primary endpoint
NCT04288687	mCRPC with DNA repair defects	Niraparib in platinum-sensitive (at least 9 weeks of platinum-based CT)	Recruiting	18	II	rPFS6
NCT04038502 (COBRA)	CRPC with homologous recombination deficiency	Carboplatin as 1st line followed by 2nd line olaparib vs. olaparib as 1st line followed by 2nd line carboplatin	Recruiting	100	II	PFS
NCT02955082 (BARCODE 2)	CRPC with germline DNA repair gene mutation	Carboplatin	Recruiting	450	II	rPFS Biochemical recurrence
NCT04592237	Aggressive variant metastatic PC	Cabazitaxel + carboplatin + cetrelimab followed by niraparib ± cetrelimab	Recruiting	120	II	PFS
NCT03263650	Aggressive variant of metastatic PC	Olaparib maintenance following cabazitaxel-carboplatin	Active, not recruiting	119	II	PFS
NCT03442556	mCRPC with homologous recombination DNA repair deficiency	Docetaxel + carboplatin + rucaparib camsylate	Not recruiting	18	II	rPFS

mCRPC, metastatic castration resistant prostate cancer; rPFS, radiological progression free survival; CT, chemotherapy

Overall, the combination of platinum-based chemotherapy with PARP inhibitors represents a novel and potentially beneficial therapeutic approach, aiming at synergistically enhancing the anti-cancer effects of both treatments and broaden the treatment opportunities for mCRPC patients. However, the identification of the optimal drug combination, as well as treatment sequence, is pivotal to minimize complications that may occur, due to drug–drug interactions and the toxicity profiles of the combined drugs, with the primary side effect being myelosuppression. Further studies to determine the dosing of individual combination agents, scheduling of treatment regimens, as well as the specific patient population, and clinical setting are required.

## Conclusion

Platinum-based chemotherapy in combination with taxanes has shown promising results in metastatic PC in a phase II study. However, the most promising activity of platinum therapy in CRPC patients is related to the presence of mutations in DNA repair genes. In this subpopulation, the use of platinum represents a viable therapeutic alternative; although, prospective and randomized studies are needed. Moreover, like other malignancies with HR mutations, the use of platinum-based chemotherapy in combination with PARP inhibitors may further enhance clinical responses in these patients with a poorer prognosis. Many aspects still need to be explored, including the optimal therapeutic sequence such as PARP-platinum-based chemotherapy or platinum-based chemotherapy-PARP, cumulative toxicity, and the most appropriate treatment setting.

## Data Availability

The datasets generated during and/or analyzed during the current study are available from the corresponding author on reasonable request.
